# On Fuzzy Positive Implicative Filters in **BE**-Algebras

**DOI:** 10.1155/2014/929162

**Published:** 2014-05-25

**Authors:** Sun Shin Ahn, Jeong Soon Han

**Affiliations:** ^1^Department of Mathematics Education, Dongguk University, Seoul 100-715, Republic of Korea; ^2^Department of Applied Mathematics, Hanyang University, Ansan 426-791, Republic of Korea

## Abstract

We study several degrees in defining a fuzzy positive implicative filter, which is a generalization of a fuzzy filter in **BE**-algebras.

## 1. Introduction

In [1], H. S. Kim and Y. H. Kim introduced the notion of a *BE*-algebra. Ahn and So [2, 3] introduced the notion of ideals in *BE*-algebras. Ahn et al. [4] fuzzified the concept of *BE*-algebras and investigated some of their properties. Jun and Ahn [5] provided several degrees in defining a fuzzy implicative filter.

In this paper, we study several degrees in defining a fuzzy positive implicative filter, which is a generalization of a fuzzy filter in *BE*-algebras.

## 2. Preliminaries

We recall some definitions and results discussed in [1–3].

An algebra (*X*; ∗, 1) of type (2, 0) is called a *BE* *-algebra* if

(BE1) *x*∗*x* = 1 for all *x* ∈ *X*,
(BE2) *x*∗1 = 1 for all *x* ∈ *X*,
(BE3) 1∗*x* = *x* for all *x* ∈ *X*,
(BE4) *x*∗(*y*∗*z*) = *y*∗(*x*∗*z*), for all *x*, *y*, *z* ∈ *X* (*exchange*).

We introduce a relation “≤” on a *BE*-algebra *X* by *x* ≤ *y*, if and only if *x*∗*y* = 1. A nonempty subset *S* of a *BE*-algebra *X* is said to be a* subalgebra* of *X*, if it is closed under the operation “∗.” By noticing that *x*∗*x* = 1, for all *x* ∈ *X*, it is clear that 1 ∈ *S*. A *BE*-algebra (*X*; ∗, 1) is said to be self-distributive, if *x*∗(*y*∗*z*) = (*x*∗*y*)∗(*x*∗*z*), for all *x*, *y*, *z* ∈ *X*.

**Definition (see [1])**

Let (*X*; ∗, 1) be a *BE*-algebra and let *F* be a nonempty subset of *X*. Then, *F* is called a* filter* of *X* if

(F1) 1 ∈ *F*,
(F2) *x*∗*y* ∈ *F* and *x* ∈ *F* imply *y* ∈ *F*,

for all *x*, *y*, *z* ∈ *X*.

A nonempty subset *F* of a *BE*-algebra *X* is called an implicative filter of *X* if it satisfies (F1) and

(F3) *x*∗(*y*∗*z*) ∈ *F* and *x*∗*y* ∈ *F* imply *x*∗*z* ∈ *F*, for all *x*, *y*, *z* ∈ *X*.

**Example (see [1])**

Let *X* : = {1, *a*, *b*, *c*, *d*, 0} be a *BE*-algebra with the following table:

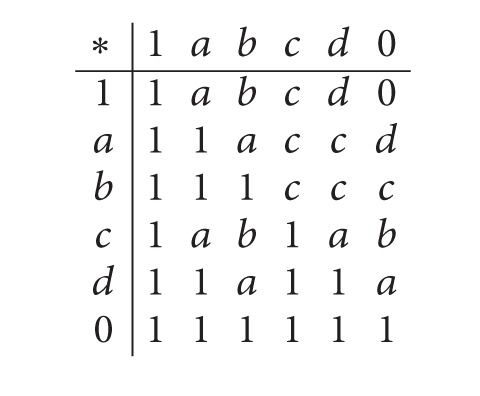
(1)
Then *F*_1_ : = {1, *a*, *b*} is a filter of *X*, but *F*_2_ : = {1, *a*} is not a filter of *X*, since *a*∗*b* ∈ *F*_2_ and *a* ∈ *F*_2_, but *b* ∉ *F*_2_.

**Proposition**

Let (*X*; ∗, 1) be a *BE*-algebra and let *F* be a filter of *X*. If *x* ≤ *y* and *x* ∈ *F*, for any *y* ∈ *X*, then *y* ∈ *F*.

**Proposition**

Let (*X*; ∗, 1) be a self-distributive *BE*-algebra. Then, the following hold, for any *x*, *y*, *z* ∈ *X*:

- *if x* ≤ *y*, *then z*∗*x* ≤ *z*∗*y and y*∗*z* ≤ *x*∗*z;*
- *y*∗*z* ≤ (*z*∗*x*)∗(*y*∗*x*)*;*
- *y*∗*z* ≤ (*x*∗*y*)∗(*x*∗*z*).

A *BE*-algebra (*X*; ∗, 1) is said to be transitive if it satisfies Proposition 4 (iii).

**Definition (see [5])**

A fuzzy subset *μ* of a *BE*-algebra *X* is called a fuzzy filter of *X*, if it satisfies, for all *x*, *y* ∈ *X*,

(d1) *μ*(1) ≥ *μ*(*x*),
(d2) *μ*(*x*) ≥ min⁡{*μ*(*y*∗*x*),  *μ*(*y*)}.

A fuzzy subset *μ* of a *BE*-algebra *X* is called a fuzzy implicative filter of *X* if it satisfies (d1) and

(d3) *μ*(*x*∗*z*) ≥ min⁡{*μ*(*x*∗(*y*∗*z*)),  *μ*(*x*∗*y*)}, for all *x*, *y*, *z* ∈ *X*.

**Definition (see [5])**

Let *F* be a nonempty subset of a *BE*-algebra *X* which is not necessarily a filter of *X*. One says that a subset *G* of *X* is an enlarged filter of *X* related to *F*, if it satisfies the following:

1. *F* is a subset of *G*,
2. 1 ∈ *G*,
3. (∀*x*, *y* ∈ *X*)(∀*x* ∈ *F*)(*x*∗*y* ∈ *F*⇒*y* ∈ *G*).

## 3. Fuzzy Positive Implicative Filters of *BE*-Algebras with Degrees in (0,1]

*X* denotes a *BE*-algebra unless specified otherwise.

**Definition**

A nonempty subset *F* of *X* is called a positive implicative filter of a *BE*-algebra *X* if it satisfies (F1) and

(F4) *x*∗((*y*∗*z*)∗*y*) ∈ *F* and *x* ∈ *F* imply *y* ∈ *F*, for all *x*, *y*, *z* ∈ *X*.

Note that every positive implicative filter of a *BE*-algebra *X* is a filter of *X*.

**Example 8**

(1) Let *X* : = {1, *a*, *b*, *c*, *d*} be a self-distributive *BE*-algebra ([1]) with the following table:

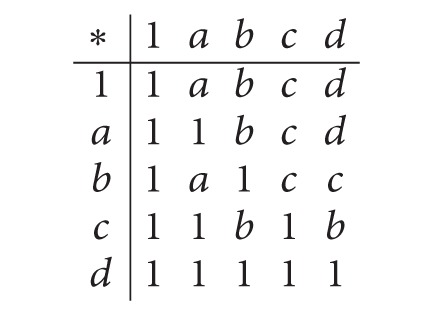
(2)
Then {1, *b*} is an implicative filter of *X* but not a positive implicative filter of *X*, since *b*∗((*a*∗*d*)∗*a*) = 1, *b* ∈ {1, *b*} and *a* ∉ {1, *b*}.

(2) Consider a *BE*-algebra *X* : = {1, *a*, *b*, *c*, *d*, 0} as in Example 2. Then {1} is a filter of *X* but not an implicative filter of *X*, since *d*∗(*a*∗0) = 1 ∈ {1},*d*∗*a* = 1 ∈ {1}, and *d*∗0 = *a* ∉ {1}. Also, it is not a positive implicative filter of *X*, since 1∗((*a*∗*b*)∗*a*) = 1 ∈ {1},1 ∈ {1} and *a* ∉ {1}.

(3) Let *X* : = {1, *a*, *b*, *c*} be a self-distributive *BE*-algebra ([5]) with the following table:

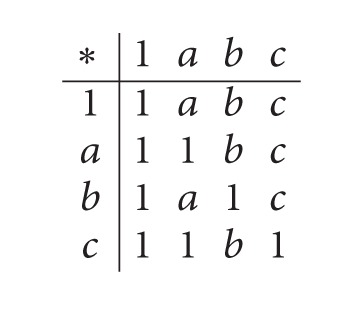
(3)
Then {1, *b*} is an implicative filter of *X* and {1, *a*, *b*} is a positive implicative filter of *X*.

**Definition**

A fuzzy subset *μ* of a *BE*-algebra *X* is called a fuzzy positive implicative filter of *X*, if it satisfies (d1) and

(d4) *μ*(*y*) ≥ min⁡{*μ*(*x*∗((*y*∗*z*)∗*y*)), *μ*(*x*)}, for all *x*, *y* ∈ *X*.

**Definition**

Let *F* be a nonempty subset of a *BE*-algebra *X* which is not necessarily a positive implicative filter of *X*. One says that a subset *G* of *X* is an enlarged positive implicative filter of *X* related to *F*, if it satisfies the following:

1. *F* is a subset of *G*,
2. 1 ∈ *G*,
3. (∀*x*, *y*, *z* ∈ *X*)(∀*x* ∈ *F*)(*x*∗((*y*∗*z*)∗*y*) ∈ *F*⇒*y* ∈ *G*).

Obviously, every positive implicative filter is an enlarged positive implicative filter of *X* related to itself. Note that there exists an enlarged positive implicative filter of *X* related to any nonempty subset *F* of *X*.

**Example**

Consider a *BE*-algebra *X* = {1, *a*, *b*, *c*, *d*} which is given in Example 8 (1). Note that *F* : = {1, *b*} is not a positive implicative filter. Then, *G* : = {1, *a*, *b*, *c*} is an enlarged positive implicative filter of *X* related to *F* and *G* is not a positive implicative filter of *X*, since *b*∗((*d*∗*d*)∗*d*) = *c* ∈ *G*,*b* ∈ *G* and *d* ∉ *G*.

**Proposition**

Let *F* be a nonempty subset of a *BE*-algebra *X*. Every enlarged positive implicative filter of *X* related to *F* is an enlarged filter of *X* related to *F*.

**Proof**

Let *G* be an enlarged positive implicative filter of *X* related to *F*. By putting *z* : = 1 in Definition 10 (3), we have

(4) (∀x,y∈X) (x∗((y∗1)∗y)=x∗y∈F,x∈F⟹y∈G).

Hence, *G* is an enlarged filter of *X* related to *F*.

The converse of Proposition 12 is not true in general as seen in the following example.

**Example**

Let *X* : = {1, *a*, *b*, *c*} be a transitive *BE*-algebra ([2]) with the following table:

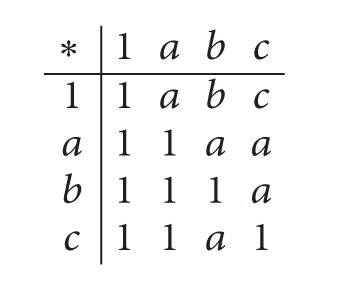
(5)
Let *F* : = {1} and *G* : = {1, *c*}. Then *G* is an enlarged filter of *F* but it is not an enlarged positive implicative filter of *F*, since 1∗((*a*∗*c*)∗*a*) = 1 ∈ *F*, 1 ∈ *F* and *a* ∉ *G*.

In what follows let *λ* and *κ* be members of (0,1], and let *n* and *k* denote a natural number and a real number, respectively, such that *k* < *n*, unless otherwise specified.

**Definition (see [5])**

A fuzzy subset *μ* of a *BE*-algebra *X* is called a fuzzy filter of *X* with degree (*λ*, *κ*), if it satisfies the following:

(e1) (∀*x* ∈ *X*)(*μ*(1) ≥ *λμ*(*x*)),
(e2) (∀*x*, *y* ∈ *X*)(*μ*(*x*) ≥ *κ*min⁡{*μ*(*y*∗*x*), *μ*(*y*)}).

A fuzzy subset *μ* of a *BE*-algebra *X* is called a fuzzy implicative filter of *X* with degree (*λ*, *κ*), if it satisfies (e1) and

(e3) (∀*x*, *y*, *z* ∈ *X*)(*μ*(*x*∗*z*) ≥ *κ*min⁡{*μ*(*x*∗(*y*∗*z*)),  *μ*(*x*∗*y*)}).

**Definition 15**

A fuzzy subset *μ* of a *BE*-algebra *X* is called a fuzzy positive implicative filter of *X* with degree (*λ*, *κ*), if it satisfies (e1) and

(e4) (∀*x*, *y*, *z* ∈ *X*)(*μ*(*y*) ≥ *κ*min⁡{*μ*(*x*∗((*y*∗*z*)∗*y*)), *μ*(*x*)}).

**Proposition (see [5])**

Every fuzzy filter of a *BE*-algebra *X* with degree (*λ*, *κ*) satisfies the following assertions:

- (∀*x*, *y* ∈ *X*)(*μ*(*x*∗*y*) ≥ *λκμ*(*y*));
- (∀*x*, *y* ∈ *X*)(*y* ≤ *x*⇒*μ*(*x*) ≥ *λκμ*(*y*));
- (∀*x*, *y*, *z* ∈ *X*)(*x* ≤ *y*∗*z*⇒*μ*(*z*) ≥ min⁡{*κμ*(*y*), *λκ* ^2^ *μ*(*x*)}).

Note that if *λ* ≠ *κ*, then a fuzzy positive implicative filter with degree (*λ*, *κ*) may not be a fuzzy positive implicative filter with degree (*κ*, *λ*) and vice versa. Obviously, every fuzzy positive implicative filter is a fuzzy positive implicative filter with degree (*λ*, *κ*), but the converse may not be true.

**Example 17**

Consider a self-distributive *BE*-algebra *X* = {1, *a*, *b*, *c*} which is given in Example 8 (3). Define a fuzzy subset *μ* : *X* → [0,1] by

(6) μ=(1abc0.70.40.80.4).

Then, *μ* is a fuzzy implicative filter of *X* with degree (2/5, 3/5) and a fuzzy filter of *X* with degree (2/5, 3/5), but it is neither a fuzzy filter of *X* nor a fuzzy positive implicative filter of *X* with degree (2/5, 3/5) since

(7) μ(1)=0.7≱0.8=μ(b),μ(a)=0.4≱0.42=35×0.7=35×μ(1)=35×min⁡{μ(1∗((a∗c)∗a))=μ(1),μ(1)}.

**Example 18**

Let *X* : = {1, *a*, *b*, *c*} be a *BE*-algebra ([2]) with the following table:

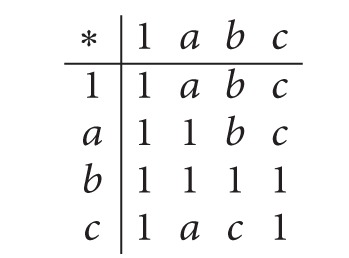
(8)
Define a fuzzy subset *μ* : *X* → [0,1] by

(9) $$\begin{array}{c} \mu =\left(\begin{array}{cccc} 1 & a & b & c\\ 0.7 & 0.8 & 0.4 & 0.4 \end{array}\right). \end{array}$$

Then, *μ* is a fuzzy positive implicative filter of *X* with degree (3/5, 2/5). But it is neither a fuzzy filter of *X* nor a fuzzy positive implicative filter of *X* with degree (2/5, 3/5) since

(10) μ(1)=0.7≱0.8=μ(a),μ(c)=0.4≱0.42=35×0.7=35×μ(1)=35×min⁡{μ(a∗((c∗b)∗c))=μ(1),μ(a)}.

Also, it is not a fuzzy implicative filter of *X* with degree (2/5, 3/5) since

(11) μ(c∗b)=μ(c)=0.4≱0.42=35×0.7=35×μ(1)=35×min⁡{μ(c∗(c∗b)) =μ(1),μ(c∗c)=μ(1)}.

**Proposition 19**

If *μ* is a fuzzy positive implicative filter of a *BE*-algebra *X* with degree (*λ*, *κ*), then *μ* is a fuzzy filter of *X* with degree (*λ*, *κ*).

**Proof**

By putting *z* : = *y* in (e4), we have

(12) μ(y)≥κmin⁡{μ(x∗((y∗y)∗y)),μ(x)}=κmin⁡{μ(x∗y),μ(x)}

for any *x*, *y* ∈ *X*. Thus, *μ* is a fuzzy filter of *X* with degree (*λ*, *κ*).

The converse of Proposition 19 is not true in general (see Example 17).

Note that a fuzzy filter with degree (*λ*, *κ*) is a fuzzy filter if and only if (*λ*, *κ*) = (1,1).

**Proposition**

Let *μ* be a fuzzy positive implicative filter of a *BE*-algebra *X* with degree (*λ*, *κ*). Then, the following holds:

(13) $$\begin{array}{c} \left(\forall x,y\in X\right)\;\left(\mu \left(x\right)\geq \kappa \lambda \mu \left(\left(x*y\right)*x\right)\right). \end{array}$$

**Proof**

Assume that *μ* is a fuzzy positive implicative filter of a *BE*-algebra *X* with degree (*λ*, *κ*) and let *x*, *y* ∈ *X*. Using (e4) and (e1), we have

(14) μ(x)≥κmin⁡{μ(1∗((x∗y)∗x)),μ(1)}=κmin⁡{μ((x∗y)∗x),λμ((x∗y)∗x)}=κλμ((x∗y)∗x).

This completes the proof.

**Proposition**

Let *μ* be a fuzzy filter of a *BE*-algebra *X* with degree (*λ*, *κ*) satisfying

(15) $$\begin{array}{c} \left(\forall x,y\in X\right)\;\left(\mu \left(x\right)\geq \mu \left(\left(x*y\right)*x\right)\right). \end{array}$$

Then, *μ* is a positive implicative filter of *X* with degree (*λ*, *κ*).

**Proof**

Let *x*, *y*, *z* ∈ *X*. Using (e2), we have

(16) μ(y)≥μ((y∗z)∗y)≥κmin⁡{μ(x∗((y∗z)∗y)),μ(x)}.

Thus, *μ* is a positive implicative filter of a *BE*-algebra *X* with degree (*λ*, *κ*).

**Corollary**

Let *μ* be a fuzzy filter of *X*. Then, *μ* is a fuzzy positive implicative filter of *X*, if and only if

(17) $$\begin{array}{c} \left(\forall x,y\in X\right)\;\left(\mu \left(x\right)\geq \mu \left(\left(x*y\right)*x\right)\right). \end{array}$$

**Proof**

It follows from Propositions 20 and 21.

**Proposition**

Every fuzzy positive implicative filter of a *BE*-algebra *X* with degree (*λ*, *κ*) satisfies the following assertions:

- (∀*x*, *y* ∈ *X*)(*μ*(*x*∗*y*) ≥ *λκμ*(*y*))*;*
- (∀*x*, *y* ∈ *X*)(*x* ≤ *y*⇒*μ*(*y*) ≥ *λκμ*(*x*)).

**Proof**

It follows from Propositions 16 and 19.

**Corollary**

Let *μ* be a fuzzy positive implicative filter of a *BE*-algebra *X* with degree (*λ*, *κ*). If *λ* = *κ*, then

- (∀*x*, *y* ∈ *X*)(*μ*(*x*∗*y*) ≥ *λ* ^2^ *μ*(*y*)),
- (∀*x*, *y* ∈ *X*)(*x* ≤ *y*⇒*μ*(*y*) ≥ *λ* ^2^ *μ*(*x*)).

**Proposition**

Let *X* be a self-distributive *BE*-algebra *X*. Let *μ* be a fuzzy positive implicative filter of *X* with degree (*λ*, *κ*). Then,

(18) $$\begin{array}{c} \left(\forall x,y\in X\right)\;\left(\mu \left(\left(y*x\right)*x\right)\geq \lambda^{2}\kappa^{3}\mu \left(y\right)\right). \end{array}$$

**Proof**

Let *x*, *y* ∈ *X*. Let *μ* be a fuzzy positive implicative filter of *X* with degree (*λ*, *κ*). By Proposition 19, *μ* is a fuzzy filter of *X* with degree (*λ*, *κ*). Since *x* ≤ (*y*∗*x*)∗*x*, using Proposition 4 (i), we have ((*y*∗*x*)∗*x*)∗*y* ≤ *x*∗*y*. Hence,

(19) (x∗y)∗y≤(y∗x)∗((x∗y)∗x)=(x∗y)∗((y∗x)∗x)≤(((y∗x)∗x)∗y)∗((y∗x)∗x).

Using Propositions 16 and 23, we have

(20) μ((y∗x)∗x) ≥min⁡{κμ(((y∗x)∗x)∗y),λκ2μ((x∗y)∗y)}  ≥min⁡{κ2λμ(y),(λκ2)κλμ(y)} =κ2λmin⁡{μ(y),λκμ(y)} =κ2λ(λκ)μ(y) =λ2κ3μ(y).

This completes the proof.

**Definition**

([6]) Let *X* be a *BE*-algebra. *X* is said to be commutative if the following identity holds:

(C) (*x*∗*y*)∗*y* = (*y*∗*x*)∗*x*; that is, *x*∨*y* = *y*∨*x*, where *x*∨*y* = (*y*∗*x*)∗*x*, for all *x*, *y* ∈ *X*.

**Theorem**

Let *X* be a commutative self-distributive *BE*-algebra. Every fuzzy positive implicative filter of *X* with degree (*λ*, *κ*) is a fuzzy implicative filter of *X* with degree (*λ*, *κ* ^3^ *λ* ^2^).

**Proof**

Let *μ* be a fuzzy positive implicative filter of *X* with degree (*λ*, *κ*). By Proposition 19, *μ* is a fuzzy filter of *X* with degree (*λ*, *κ*). Using (BE4) and Proposition 4 (iii), we obtain (*x*∗(*y*∗*z*))∗((*x*∗*y*)∗(*x*∗(*x*∗*z*))) = 1, for any *x*, *y*, *z* ∈ *X*. Hence, by Proposition 16 (iii), we have *μ*(*x*∗(*x*∗*z*)) ≥ min⁡{*κμ*(*x*∗(*y*∗*z*)), *λκ* ^2^ *μ*(*x*∗*y*)}. On the other hand, using (BE4) and (C), we obtain

(21) ((x∗z)∗z)∗(x∗z)=x∗(((x∗z)∗z)∗z)=x∗((z∗(x∗z))∗(x∗z))=x∗(1∗(x∗z))=x∗(x∗z).

Using Proposition 20, we have

(22) μ(x∗z)≥κλμ(((x∗z)∗z)∗(x∗z))=κλμ(x∗(x∗z))≥κλmin⁡{κμ(x∗(y∗z)),λκ2μ(x∗y)}=κ2λmin⁡{μ(x∗(y∗z)),κλμ(x∗y)}≥κ3λ2min⁡{μ(x∗(y∗z)),μ(x∗y)}.

This completes the proof

Denote by *F*_PI_(*X*) the set of all positive implicative filters of a *BE*-algebra *X*. Note that a fuzzy subset *μ* of a *BE*-algebra *X* is a fuzzy positive implicative filter of *X*, if and only if

(23) $$\begin{array}{c} \left(\forall t\in \left[0,1\right]\right)\;\left(U\left(\mu ;t\right)\in F_{\text{PI}}\left(X\right)\cup \left\{\emptyset \right\}\right). \end{array}$$

But we know that, for any fuzzy subset *μ* of a *BE*-algebra *X*, there exist *λ*, *κ* ∈ (0,1) and *t* ∈ [0,1] such that

1. *μ* is a fuzzy positive implicative filter of *X* with degree (*λ*, *κ*),
2. *U*(*μ*; *t*) ∉ *F*_PI_(*X*)∪{*∅*}.

**Example**

Consider a *BE*-algebra *X* = {1, *a*, *b*, *c*, *d*} which is given in Example 8 (1). Define a fuzzy subset *μ* : *X* → [0,1] by

(24) $$\begin{array}{c} \mu =\left(\begin{array}{ccccc} 1 & a & b & c & d\\ 0.4 & 0.3 & 0.5 & 0.3 & 0.3 \end{array}\right). \end{array}$$

If *t* ∈ (0.4,0.6], then *U*(*μ*; *t*) = {1, *b*} is not a positive implicative filter of *X*, since *b*∗((*a*∗*d*)∗*a*) = 1, *b* ∈ {1, *b*}, and *a* ∉ {1, *b*}. But *μ* is a fuzzy positive implicative filter of *X* with degree (0.4,0.6).

**Theorem**

Let *μ* be a fuzzy subset of a *BE*-algebra *X*. For any *t* ∈ [0,1] with *t* ≤ max⁡{*λ*, *κ*}, if *U*(*μ*; *t*) is an enlarged positive implicative filter of *X* related to *U*(*μ*; *t*/max⁡{*λ*, *κ*}), then *μ* is a fuzzy positive implicative filter of *X* with degree (*λ*, *κ*).

**Proof**

Assume that *μ*(1) < *t* ≤ *λμ*(*x*), for some *x* ∈ *X* and *t* ∈ (0, *λ*]. Then *μ*(*x*) ≥ *t*/*λ* ≥ *t*/max⁡{*λ*, *κ*} and so *x* ∈ *U*(*μ*; *t*/max⁡{*λ*, *κ*}); that is, *U*(*μ*; *t*/max⁡{*λ*, *κ*}) ≠ *∅*. Since *U*(*μ*; *t*) is an enlarged filter of *X* related to *U*(*μ*; *t*/max⁡{*λ*, *κ*}), we have 1 ∈ *U*(*μ*; *t*); that is, *μ*(1) ≥ *t*. This is a contradiction, and thus *μ*(1) ≥ *λμ*(*x*), for all *x* ∈ *X*.

Now suppose that there exist *a*, *b*, *c* ∈ *X* such that *μ*(*b*) < *κ*min⁡{*μ*(*a*∗((*b*∗*c*)∗*b*)), *μ*(*a*)}. If we take *t* : = *κ*min⁡{*μ*(*a*∗((*b*∗*c*)∗*b*)), *μ*(*a*)}, then *t* ∈ (0, *κ*]⊆(0, max⁡{*λ*, *κ*}]. Hence, *a*∗((*b*∗*c*)∗*b*) ∈ *U*(*μ*; *t*/*κ*)⊆*U*(*μ*; *t*/max⁡{*λ*, *κ*}) and *a* ∈ *U*(*μ*; *t*/*κ*)⊆*U*(*μ*; *t*/max⁡{*λ*, *κ*}). It follows from Definition 10 (3) that *b* ∈ *U*(*μ*; *t*) so that *μ*(*b*) ≥ *t*, which is impossible. Therefore,

(25) $$\begin{array}{c} \mu \left(y\right)\geq \kappa \min\left\{\mu \left(x*\left(\left(y*z\right)*y\right)\right),\mu \left(x\right)\right\} \end{array}$$

for all *x*, *y*, *z* ∈ *X*. Thus, *μ* is a fuzzy positive implicative filter of *X* with degree (*λ*, *κ*).

**Corollary**

Let *μ* be a fuzzy subset of a *BE*-algebra *X*. For any *t* ∈ [0,1] with *t* ≤ *k*/*n*, if *U*(*μ*; *t*) is an enlarged positive implicative filter of *X* related to *U*(*μ*; (*n*/*k*)*t*), then *μ* is a fuzzy positive implicative filter of *X* with degree (*k*/*n*, *k*/*n*).

**Theorem**

Let *t* ∈ [0,1] be such that *U*(*μ*; *t*)  (≠*∅*) is not necessarily a positive implicative filter of a *BE*-algebra *X*. If *μ* is a fuzzy positive implicative filter of *X* with degree (*λ*, *κ*), then *U*(*μ*; *t*min⁡{*λ*, *κ*}) is an enlarged positive implicative filter of *X* related to *U*(*μ*; *t*).

**Proof**

Since *t*min⁡{*λ*, *κ*} ≤ *t*, we have *U*(*μ*; *t*)⊆*U*(*μ*; *t*min⁡{*λ*, *κ*}). Since *U*(*μ*; *t*) ≠ *∅*, there exists *x* ∈ *U*(*μ*; *t*) and so *μ*(*x*) ≥ *t*. By (e1), we obtain *μ*(1) ≥ *λμ*(*x*) ≥ *λt* ≥ *t*min⁡{*λ*, *κ*}. Therefore, 1 ∈ *U*(*μ*; *t*min⁡{*λ*, *κ*}).

Let *x*, *y*, *z* ∈ *X* be such that *x*∗((*y*∗*z*)∗*y*) ∈ *U*(*μ*; *t*) and *x* ∈ *U*(*μ*; *t*). Then *μ*(*x*∗((*y*∗*z*)∗*y*)) ≥ *t* and *μ*(*x*) ≥ *t*. It follows from (e4) that

(26) μ(y)≥κmin⁡{μ(x∗((y∗z)∗y)),μ(x)}≥κt≥tmin⁡{λ,κ};

so that *y* ∈ *U*(*μ*; *t*min⁡{*λ*, *κ*}). Thus, *U*(*μ*; *t*min⁡{*λ*, *κ*}) is an enlarged positive implicative filter of *X* related to *U*(*μ*; *t*).
